# Antifungal Activity of the Sesquiterpene Lactones from *Psephellus bellus*

**DOI:** 10.3390/plants10061180

**Published:** 2021-06-09

**Authors:** Joanna Nawrot, Zygmunt Adamski, Beata Kamińska-Kolat, Honorata Kubisiak-Rzepczyk, Anna Kroma, Gerard Nowak, Justyna Gornowicz-Porowska

**Affiliations:** 1 Department and Division of Practical Cosmetology and Skin Diseases Prophylaxis, Poznan University of Medical Sciences, Mazowiecka 33, 60-623 Poznan, Poland; joannac@ump.edu.pl (J.N.); beata.kaminska@oriflame.com (B.K.-K.); kromkaania@interia.pl (A.K.); gnowak.gerard@gmail.com (G.N.); 2 Department of Dermatology, Poznan University of Medical Sciences, Przybyszewskiego 49, 60-356 Poznan, Poland; adamskiz@poczta.onet.pl; 3 Department of Dermatology and Venereology, Poznan University of Medical Sciences, Przybyszewskiego 49, 60-356 Poznan, Poland; rzepczykh@poczta.onet.pl

**Keywords:** sesquiterpene lactones, antifungal activity, *Psephellus bellus*

## Abstract

Due to increasing resistance of pathogenic fungi to antifungal treatments, new types of drugs are needed. For this purpose, active substances with antifungal properties occurring in natural compounds should be considered. The herb *Psephellus bellus* shows strong antifungal activity and is characterized by unique guaianolides, which have an ester on C-2. Thus, a specialized method of isolation and testing was applied to assess the pharmacological effects of these guaianolides. After phytochemical analysis (chromatography and spectral methods), selected lipophilic compounds and the herb extract of this species containing 26 sesquiterpene lactones were tested. The antifungal effect of the herbal compounds was determined on clinical strains of fungi *Candida*, *Rhodotorula*, *Trichophyton*, *Microsporum*, and *Scopulariopsis* using a diffusion test. The MTT assay was employed to study the cytotoxic effects of the extract against human fibroblasts. Statistical analysis was performed. All analyzed compounds exhibited antifungal activity in cultivations suitable for assessment. Most lipophilic cebellins from *Psephellus bellus* prevent the growth of most fungal strains.

## 1. Introduction

Fungal infections affect about 40% of the world’s population and may be viewed as an epidemiological, therapeutic, and social problem. Commonly used antibiotics change people’s microbial flora, which has caused an increase in the number of people with impaired immunity. Factors such as industrial development, agriculture, technology, and the increase in the duration of people’s lives have increased the susceptibility of the population to infections. The number and variety of fungi causing diseases are increasing globally. In different parts of the world, sometimes even within one country, differences exist in fungal flora regarding the frequency of occurrence of individual species [1,2,3]. Moreover, pathogenic fungi are becoming resistant to commonly used and widely known antifungal treatments. Hence, novel active pharmaceutical ingredients are needed, including those with novel mechanisms of action or that act on new receptors. Several natural products exhibit promising antifungal properties, and these compounds might provide new active substances with the desired activity. In this context, compounds such as sesquiterpene lactones and coumarins, known for their strong antimicrobial effects, have often been studied for their antifungal properties [4,5,6]. Numerous investigations showed that plant extracts combined with antibiotics increase their activity, and decrease the required doses of antibiotics and their side effects. These positive interactions are considered a potential strategy to combat bacterial resistance [7].

It has been proven that these properties of sesquiterpene lactones are enabled by their specific skeletal structure: a lactone ring coupled with an exo-methylene, which results in the inhibition of cellular enzymes through Michael nucleophilic addition [8]. The substituents, mainly esters and hydroxyl groups, and their conformation, are responsible for compound potency and bioavailability by making them either lipophilic or hydrophilic. The lipophilicity of compounds allows them to penetrate cell walls more efficiently; this phenomenon is described as the lipophilicity rule [4].

Sesquiterpene lactones are the most characteristic compounds occurring in the plants of the *Centaureinae* subtribe (*Compositae*). Guaianolides are the most commonly appearing type of lactones [9]. Either in isolated form or extracts, they show broad biological properties, including anti-inflammatory, antibacterial, antiviral (including against SARS-CoV-2) [10], antihelminthic [11], and antimigraine [12] activities.

**Figure 1:**
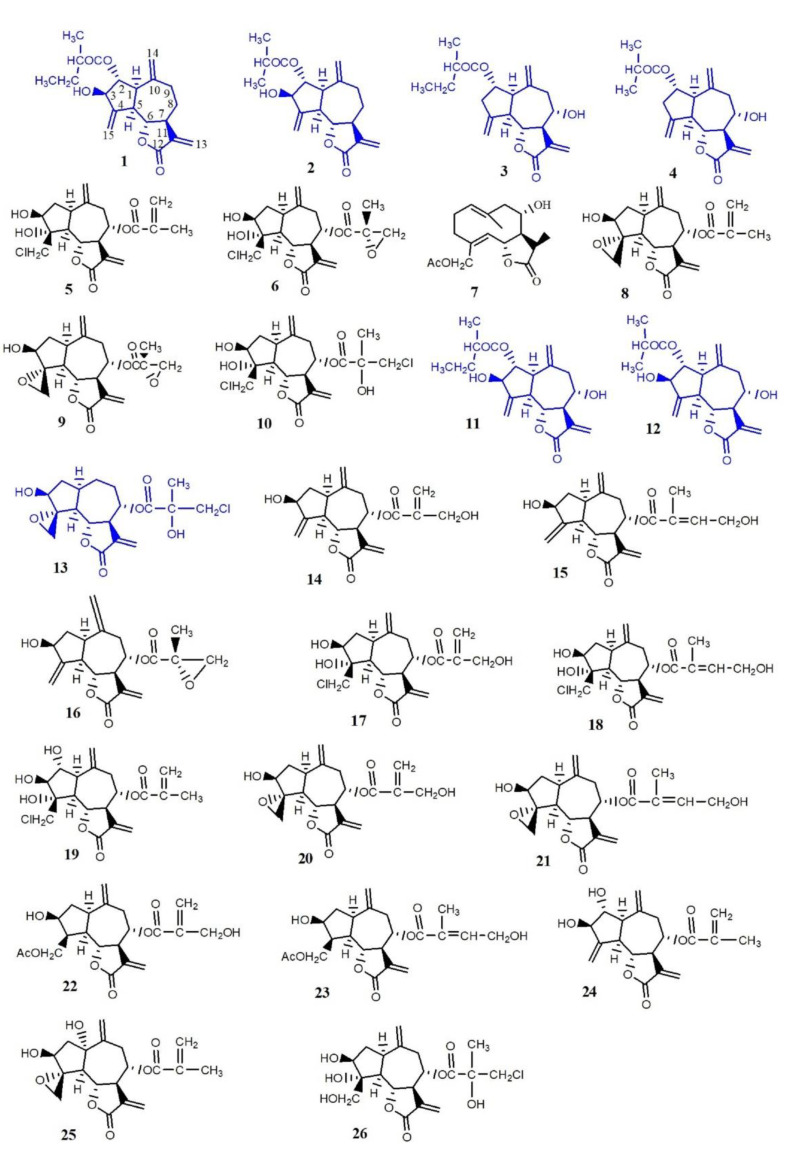
Chemical structures of compounds with features responsible for their antifungal properties: **1**. cebellin L, **2**. cebellin O, **3**. cebellin K, **4**. cebellin N, **5**. 19-deoxychlorojanerin, **6**. 17,18-epoxy-19-deoxychlorojanerin, **7**. cebellin M, **8**. 8-desacylo-8α-(2′-methyl-acryloxy)-subluteolide, **9**. repin, **10**. centaurepensin, **11**. cebellin A, **12**. cebellin B, **13**. acroptilin, **14**. cynaropicrin, **15**. cebellin F, **16**. 15-deoxyrepin, **17**. cebellin C, **18**. cebellin D, **19**. cebellin E, **20**. janerin, **21**. 8α-4′-tiglinate-8-desacetyl-subluteolide, **22**. cebellin G, **23**. cebellin H, **24**. cebellin I, **25**. repdiolide, **26**. cebellin J. Compounds chosen for antifungal study.

There are at least 26 different sesquiterpene lactones (1–26) in *Psephellus bellus* (Figure 1) [13,14], among which 10 guaianolides occur only in this species and those related to it (cebellins: L, O, K, N, A, B, G, H, I, and J) [15].

Cebellins L (**1**), O (**2**), K (**3**), N (**4**), A (**11**), and B (**12**) all have an ester on C-2. The characteristic features in the structure of these four guaianolides distinguish them from compounds occurring in the other *Centaureinae* plants. They either lack the hydroxyl group on C-3 (compounds **3** and **4**) or the substituent on C-8 (compounds **1** and **2**). These features are responsible for the lipophilicity of compounds **1**–**4**. Moreover, the presence of ester groups on C-2 with a methylene substituent also increases their lipophilicity.

The other nine sesquiterpene lactones from *P. bellus* are also lipophilic: 19-deoxychlorojanerin (**5**), 17,18-epoxy-19-deoxychlorojanerin (**6**), cebellin M (**7**), 8-desacyloxy-8α-(2′-methyloaryloxy)-subluteolide (**8**), repin (**9**), centaurepensin (**10**), cebellin A (**11**), cebellin B (**12**), and acroptilin (**13**).

The sesquiterpenoids showing interesting antifungal activities are reported in the literature [16]. Their mechanism of action is not fully understood, although it was suggested to involve membrane disruption due to their lipophilic nature [17].

Antifungal studies of various sesquiterpene lactones showed that the most effective compounds are those that contain an α-methyleno-γ-lactone group but lack bulky sterically inhibitory groups, which limit access to the α-methyleno-γ-lactone. Nonpolar or weakly polar compounds were found to be more bioactive, and sesquiterpene lactones of a guaianolide structure had the greatest antifungal potency [18,19]. As such, there was reason to think that the lipophilic compounds from the herb *P. bellus* may have antifungal properties, which was our motivation to conduct the appropriate studies, as presented in this paper.

For this study, compounds **1** and **11**–**13** were chosen, in addition to the mixture of compounds **2–4** and the extract of the herb of *P. bellus* containing 26 sesquiterpene lactones. Their antifungal effect was tested on four clinical strains of yeast-like fungi, four clinical strains of dermatophytes, and one clinical strain of mold fungus.

## 2. Results and Discussion

### 2.1. Isolation and Identification of Compounds from P. bellus for Antifungal Studies

Four guaianolides were isolated from a methylene chloride (CH_2_Cl_2_) extract of *P. bellus* and identified using their ^1^H NMR spectra (Table 1) to study their antifungal properties. Compound **1** contains an additional proton at C-8 (δ = 2.24 ppm). Compounds **1, 11,** and **12** with a substituent at C-2 are characterized by signals of proton H-3α and H-2β (dd 4.94–4.98 ppm). The H-8 signal indicates the presence of free hydroxyl in compounds **11** and **12** at C-8 (δ = 4.01 ppm).

**Figure 2:**
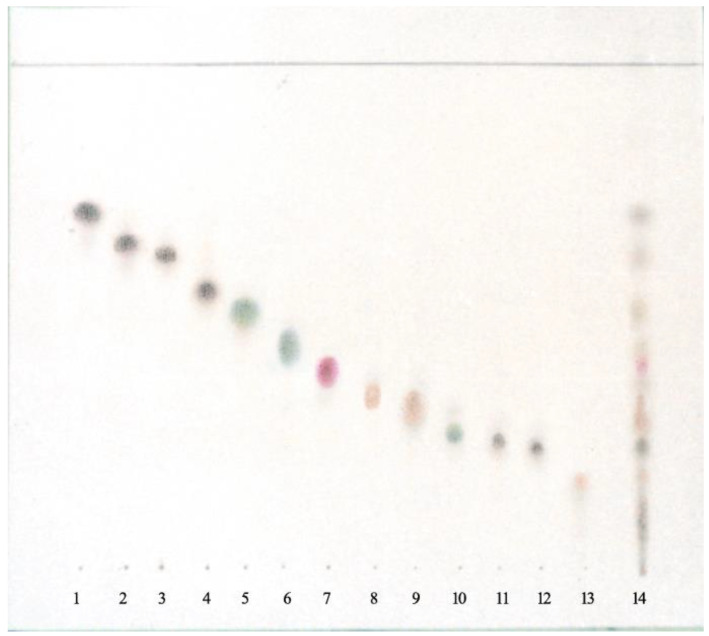
TLC of lipophilic compounds from *Psephellus bellus*. Adsorbent: silica gel. Mobile phase: n-hexane–CH_2_Cl_2_–AcOEt 4:2:5. **1**. cebellin L, **2**. cebellin O, **3**. cebellin K, **4**. cebellin N, **5**. 19-deoxychlorojanerin, **6**. 17,18-epoxy-19-deoxychlorojanerin, **7**. cebellin M, **8**. 8-desacylo-8α-(2′-methyl-acryloxy)-subluteolide, **9**. repin, **10**. centaurepensin, **11**. cebellin A, **12**. cebellin B, **13**. acroptilin, and **14**. extract from *Psephellus bellus* herb.

The OH group signal at C-8 of compounds **11** and **12** was observed at 1.85 ppm. The characteristic signals indicated the presence of the 4α,15-epoxy group in compound **13** of oxirane protons (doublets of H-15 and H-15’ at δ = 3.34 and 3.07 ppm with J (15, 15’) = 4.2 Hz) [12,13,14,16,17,20]. The mixture of compounds **2**–**4** was identified using the TLC method (Figure 2).

### 2.2. Antifungal Activity of the Guaianolides from P. bellus

**Figure 3:**
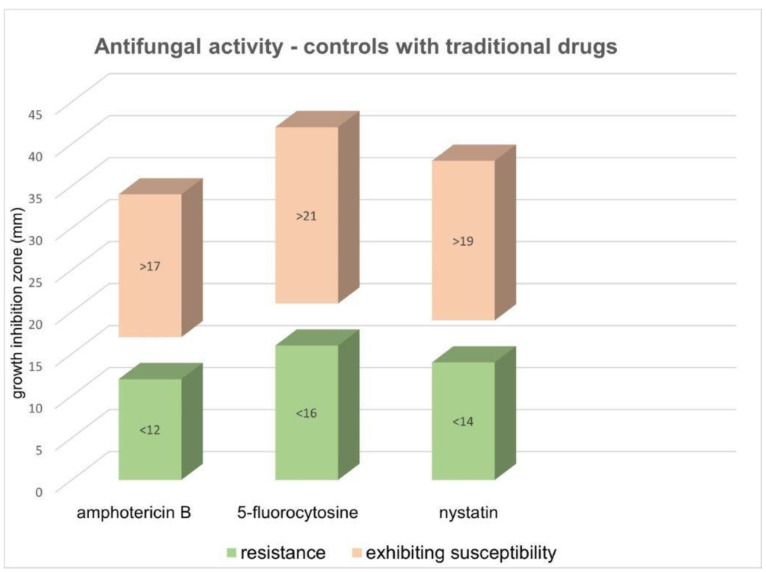
Antifungal activity of controls with traditional drugs for *C. albicans*. The results are expressed as growth inhibition for three antifungal drugs.

No inhibition zone was observed in the negative controls. The results (diameter of growth inhibition zone) of controls with traditional drugs *(C. albicans)* were as follows (Figure 3): (i) amphotericin B, exhibiting susceptibility >17 mm, resistance <12 mm; (ii) 5-fluorocytosine, exhibiting susceptibility >21 mm, resistance <16 mm; and (iii) nystatin, exhibiting susceptibility >19 mm, resistance <14 mm.

The analysis of all suitable-for-assessment fungi cultures showed that all of the studied compounds have antifungal properties. Because of the limited amount of some compounds, only 0.39% solutions of the compounds and extract were used during the study.

The diameters of the inhibition zones were measured to assess the susceptibility of the fungi to the activity of the tested compounds. The analyzed fungi were classified into the following categories: (i) susceptible, with an inhibition zone diameter over 10 mm; (ii) moderate-susceptible, with an inhibition zone diameter between 1 and 10 mm; and (iii) resistant, with no inhibition zone.

The antifungal activity investigation of the examined compounds from *P. bellus* was performed in duplicate and the inhibition zones were measured 7–10 times each. Detailed statistical analysis results and a comparison of the antifungal activity *P. bellus* compounds in relation to fungal strains are presented in Table 2 and Table 3. The measurements were recorded over 3 days from *Candida* cultures, and 14 days from the dermatophytes and mold fungus (*Scopulariopsis brevicaulis*).

The examined compounds and *P. bellus* extract inhibited the growth of *Candida albicans* fungi less than *Candida non-albicans*. However, due to the lack of growth of *C. albicans*, the antifungal activity of this strain could only be assessed for the two substances. *C. albicans* was sensitive only to the mixture of K + N + O (**2**–**4**) cebellins (the zone of growth inhibition was 12 mm). Cebellin A (**11**) showed a significantly weaker inhibitory effect on the growth of *C. albicans* (the zone of growth inhibition was 2 mm).

In comparison to *C. albicans,* the strains were easier to grow, which is why the antifungal activity of all the studied compounds was observed.

Most fungi (i.e., *Candida albicans*, *Microsporum canis*, and *Rhodotorula rubra*) strains were susceptible to cebellins **1–4** from *P. bellus*. These compounds are the most lipophilic of the studied lactones. Their lipophilicity enables those cebellins to penetrate fungus cell walls and destroy them more easily [4].

Of the yeast-like fungi, *Candida famata* and *C. glabrata*, in addition to dermatophytes from *Trichophyton* genus *T. rubrum* and *T. mentagrophytes* var. *interdigitale*, were the most susceptible to the analyzed compounds. The multiple comparison test showed a statistically significant difference in the susceptibility to the analyzed compounds between *Scopulariopsis brevicaulis* and *T. mentagrophytes* var. *interdigitale*, and between *Scopulariopsis brevicaulis* and *C. glabrata*. The highest potency of the *P. bellus* herb extract was shown by an inhibition zone diameter that reached 34 mm.

The findings suggest that lipophilic sesquiterpene lactones (compounds **1**–**13**), mostly those with an ester on C-2 (compounds **1**–**4**, **11**, **12**), are responsible for the antifungal effect of the extract from *P. bellus*. The antifungal studies of various sesquiterpene lactones showed that sesquiterpene lactones of a guaianolide structure have the strongest antifungal potency. Moreover, the highest potency of the studied extract suggested that guaianolides present in the extract show a synergistic effect.

**Figure 4:**
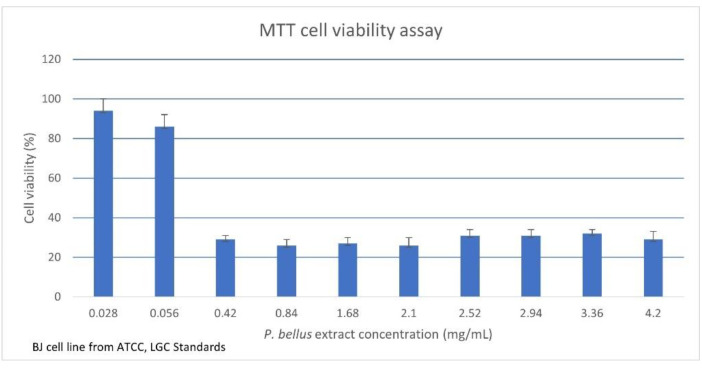
The MTT cell viability assay on a human skin fibroblast cell line. The results are expressed as cell viability with different *P. bellus* extract concentrations. Values are given as mean + standard error.

Because all examined compounds and full extract were analyzed in relation to clinical fungal strains (especially dermatophytes), we decided to evaluate biosafety (toxicity) on human skin cells using the MTT test. The cell viability determined by the MTT assay was 94% for 0.028 mg/mL and 29% for 4.2 mg/mL of *P. bellus* extract. The IC50 value was calculated as 0.139 mg/mL. The obtained cell viability results for each *P. bellus* extract concentration are shown in Figure 4. However, we observed that the extract precipitates crush the cells, causing mechanical damage. Furthermore, phase contrast microscopy of cultured cells exposed to the *P. bellus* extract suggested that the cells did not retain a confluent appearance and were damaged, probably due to the weight of the sample. As such, we could not directly reach conclusions about the biosafety of *P. bellus* extract; further investigation with micronized extract is needed. Moreover, the full extract of *P. bellus* may be relatively more toxic than the single test compounds. Overall, the findings show that the combined compounds in full *P. bellus* extract do not act in synergy to protect or rejuvenate the cells. Thus, in the next step, the cytotoxicity effect of the individual compound should be determined.

In our study, the biological activities of natural compounds of *P. bellus* were investigated in relation to the antifungal properties, revealing novel potential application in therapy. These novel inhibitors—sesquiterpene lactones, guaianolides, and coumarins—may be potentially therapeutic against fungal infections. Notably, the frequency of fungal infections is still increasing [21] while resistance of pathogenic fungi to classical antifungal agents is increasing and the number of available drugs is decreasing [22]. As such, identifying and commercializing alternative, active substances from natural sources as antifungal remedies are required [23,24,25]. To the best of our knowledge, this is the first report on the antifungal activity of various compounds of *P. bellus*.

## 3. Materials and Methods

The bioethics committee approved the study protocol under Act 1188/17, based on Polish legislation and Good Clinical Practices at the Poznań University of Medical Sciences.

### 3.1. Plant Material

**Figure 5:**
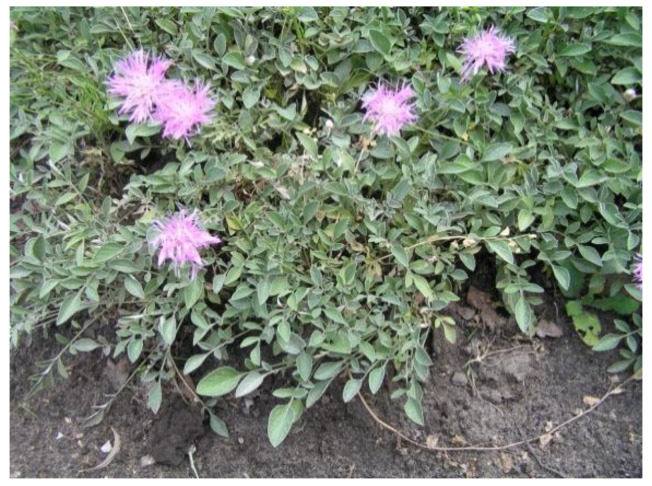
*Psephellus bellus* in the Garden of Department and Division of Practical Cosmetology and Skin Diseases Prophylaxis.

Herbs of *Psephellus bellus* (Trautv.) Wagenitz (syn. *Centaurea bella* Trautv.) were collected from the Botanical Garden of the Department and Division of Practical Cosmetology and Skin Diseases Prophylaxis, University of Medical Sciences (Poznań, Poland) (Figure 5), where voucher specimens (voucher numbers 21/1990 and 55/2014) are deposited. Seeds of *P. bellus* were provided by the Botanical Garden in Karaganda (Kazachstan) in accordance with the Convention on Biological Diversity from Rio de Janeiro 1992. The seeds were all gathered in the same year from their natural habitat on the Caucasus Mountains.

The dried herbs of *P. bellus* (758 g) were crushed and soaked in 4.5 L MeOH 3 times at room temperature. The MeOH extract was evaporated under reduced pressure and lyophilized to obtain 5.5852 g (0.74%). We used 25.21 mg of extract for the antifungal studies, and the remaining was dissolved in 0.5 L H_2_O. The aqua solution was re-extracted 3 times with 0.25 L CH_2_Cl_2_. The CH_2_Cl_2_ extract was dried with anhydrous Na_2_SO_4_, filtrated, and evaporated, producing a residue (3.8 g). It was used as the material for phytochemical studies.

The compounds were separated by column chromatography (CC) on silica gel (particle size: 0.063–0.200 mm; Merck, Darmstadt, Germany; Art. 7733). The column was run by gravity flow with CH_2_Cl_2_, and a mixture of CH_2_Cl_2_ and (CH_3_)_2_CO (ratio 35:1) as the eluent. The polarity was gradually increased by adding (CH_3_)_2_CO. Selected fractions were further rechromatographed on silica gel (particle sizes of <0.063 mm; Merck, Darmstadt, Germany; Art. 7729) with CH_2_Cl_2_–n-hexane–(CH_3_)_2_CO–AcOEt (ratio 8:3; 1:1) and n-hexane–AcOEt (1:2; 1:5, and 1:8). The NMR spectra were run on a Bruker Avance 600 instrument using 600 and 150 MHz frequencies for hydrogen nuclei (^1^H) and carbon nuclei (^13^C), respectively, and tetramethylsilane (TMS) was used as the internal standard. The spectra were obtained for CDCl_3_, CD_3_OD, or DMSO-d_6_ solutions at 298 K.

### 3.2. Antifungal Susceptibility Study

All strains were isolated from the infected skin and nails of patients of the Department of Dermatology, Poznań University of Medical Sciences (Microbiology Laboratory). The fungal isolates were identified according to microscopic examination and visual observation on different cultivation media. Strains were correctly identified using an API ID 32^®^ (bioMerieux, Lyon, France).

The Kirby–Bauer disk diffusion susceptibility test was used to determine the antifungal activity of the examined substances or drugs and the growth inhibition zone [17]. The diameter of the inhibition zones was measured to assess the antifungal properties of the studied substances. The inhibition zones were measured in millimeters.

Clinical strains of *Candida albicans, C. famata, C. glabrata, C. parapsilosis, Rhodotorula rubra, Trichophyton rubrum, T. mentagrophytes var. interdigitale, Microsporum canis,* and Scopulariopsis brevicaulis were suspended in 0.9% NaCl solution. Then, 10 mL of the suspension of all examined fungal strains was adjusted to 1 McFarland unit by a densitometer and were poured onto 9 cm agar plates with the following media: (i) 0.5% yeast nitrogen base (YNB; Difco, 3% glucose, and 1.8% agar; pH 7) for yeast strains; (ii) RPMI (bioMèrieux, Marcy l’Etoile, France) for dermatophytes and mold fungi.

The microorganism suspension was poured onto the plate containing YNB or RPMI agar and then small (0.5 mm), sterile paper discs impregnated with 10 µg of antifungal agent (0.39%) were placed on the plates. The measurement was made in duplicate, thus two plates were used for a single investigation. The substance then diffused from the filter into the medium and inhibited the growth of the surrounding microorganisms. The inhibition zone was measured visually under a stereomicroscope (Nikon SMZ800, Tokyo, Japan). All examinations were conducted simultaneously by two independent observers with microbiology experience (especially medical mycology), who were blinded to the previous results.

After 15 min, the Petri dishes were incubated. *Candida* cultures were incubated for 72 h at 36 °C. Dermatophytes (*Trichophyton* sp., *Rhodotorula* sp., and *Microsporum* sp.) and mold fungus (*Scopulariopsis brevicaulis*) were incubated at 27 °C for two weeks.

Two culture media were used: RPMI agar (bioMèrieux, Marcy l’Etoile, France) and 0.5%, YNB agar (Difco; glucose 3%, agar 1.8%, pH 7) obtained from DHN (Cracow, Poland). Then 15 mL of YNB/RPMI was poured into a 9 cm Petri dish. The sterilization was conducted under 1.5 atm pressure for 15 min.

We impregnated 5 mm filter paper disks with the examined compounds. Sterile NaCl 0.9% was used as the negative control.

As positive controls, we determined the sensitivity of *C. albicans* strains to amphotericin B (2 µg/mL), 5-fluorocytosine (16 µg/mL), and nystatin (1.25 µg/mL) through the disk diffusion susceptibility test using 5 mm filter paper disks impregnated with the mentioned commercially available drugs obtained from DHN (Cracow, Poland).

We decided not to establish the minimum inhibitory concentration (MIC) of each substance (no references were available to determine the MIC of the examined compounds). Due to the lack of standards of the used experimental materials, we could not assess the MIC through in vitro trials. Moreover, reported data indicated [18] that the MIC is of little value without the corresponding ability to interpret its clinical meaning. Certain authors [26] suggested that for some clinical isolates, MIC reading is complicated by the occurrence of MIC phenomena. It is known that the observed trailing and paradoxical effects (PXE), e.g., in *C. albicans*, complicate the unambiguous and reproducible determination of MICs. MICs are not a physical measurement. Moreover, host factors play a critical role in determining clinical outcomes. As such, dermatological clinicians decided not to conduct MIC testing.

### 3.3. Cytotoxicity Experiments

The MTT assay was employed to measure the cytotoxic effects of *P. bellus* extract on human dermal fibroblast (the BJ cell line from ATCC, LGC Standards; ATCC^®^ CRL 2522™). Human dermal fibroblasts were cultured in EMEM supplemented with 5% fetal bovine serum, 4 mM L-glutamine, and 1% penicillin–streptomycin. Briefly, cells were seeded into flat-bottomed 96-well cell culture plates. The cells were then treated with varying concentrations of *P. bellus* extract. Dimethyl sulfoxide (DMSO) was used as a solvent. Appropriate controls (1% SDS and incubation only in culture medium) were used. The stocks of different dilutions were prepared (0.028–4.2 mg/mL) and each concentration of 100 µL was added in six repetitions to the respective wells. The plate was incubated at 37 °C in a humidified 5% CO_2_ incubator. Non-treated control cells were also maintained for comparing growth inhibition. The entire plate was observed after 24 h of treatment using a contrast tissue culture microscope to identify any detectable variations in the morphology of the cells.

The sample content in the wells was removed after 24 h of incubation and rinsed with phosphate-buffered saline (PBS) with calcium and magnesium ions. Subsequently, 100 µL of reconstituted MTT solution (0.5 mg/mL) was placed in all test and control wells. The plate was incubated for 3 h at 37 °C in a CO_2_ incubator.

The MTT was removed and 100 µL of isopropanol was added. Then, the wells were mixed for 15 min to solubilize the insoluble formazan crystals.

The absorbance values were measured at a wavelength of 570 nm with a microplate reader (TECAN Spark 10M, Zürich, Switzerland). The IC50 value was calculated.

### 3.4. Statistical Analysis

Statistical analysis was performed using STATISTICA version 13 (StatSoft, Inc., Tulsa, OK, USA). One-way ANOVA with a post-test (Tukey test) was used to identify possible significant differences between the compound effect on growth inhibition of examined fungal strains. The mean value of growth inhibition with the standard deviation was calculated. A value of *p* < 0.05 was considered statistically significant.

## 4. Conclusions

Containing compounds with possible antifungal properties, *P. bellus* is a plant with significant chemical and pharmacological potential.

The findings of the biological study described in this paper suggest that C-2 ester guaianolides from *P. bellus* are responsible for the potent antifungal activity of the extract from this plant. Pathogenic fungi are becoming resistant to commonly used and widely known antifungal treatments; thus, compounds from the studied material from *P. bellus* might provide a promising new source of compounds to be further evaluated in future studies for use as new antifungal agents in the clinic. Such a drug may be used for the treatment of infections caused by *T. mentagrophytes* var. *interdigitale*. It is possible that the drug would also affect infectious fungi strains that are resilient to the already available antifungal preparations.

Natural plant products need to be standardized and preliminary studies conducted to evaluate the possible risks, such as toxicity, of these compounds.

## Figures and Tables

**Table 1:** ^1^H NMR (600 MHz) spectroscopic data (δ_H_ in ppm, mult; *J* in Hz) of compounds: **1** and **11–13**.

Pos.	1 ^a^	11 ^a^	12 ^a^	13 ^b^
1	2.93 dd (6.5; 9.8)	2.98 m	2.98 m	3.38 m
2α	-	-	-	2.51 m
2β	4.94 dd (6.5; 5.8)	4.98 dd (9.3; 7.3)	4.96 dd (9.0; 7.2)	1.81 m
3	4.41 m	4.43 m	4.43 m	3.99 m
5	3.01 t	2.98 m	2.85 m	2.02 d (11.3)
6	4.16 dd (9.0; 9.8)	4.16 dd (10.6; 9.0)	4.25 dd (10.1; 9.0)	4.08 d 9.3)
7	2.88 m	2.81 m	3.19 m	3.08 t (9.4; 3.5; 3.2)
8α	2.25 m	4.01 m	4.01 dd (3.0; 5.3)	5.24 m
8β	2.24 m	-	-	-
9α	2.25 m	2.32 dd (5.4; 14.6)	2.37 dd (5.3; 14.6)	2.49 dd (15.2; 2.7)
9β	2.47 m	2.70 dd (14.6; 5.4)	2.70 dd (14.6; 5.3)	2.71 dd (15.2; 5.2)
13	6.25 d (3.6)	6.29 dd (3.5; 0.8)	6.21 d (0.8)	6.24 d (1.6)
13′	5.52 d (3.2)	6.16 dd (3.2; 0.8)	6.16 d (0.8)	5.57 d (1.6)
14	4.98 d (2.5)	5.11 d (1.6)	5.14 d (1.7)	5.21 d (1.8)
14′	4.93 d (2.1)	5.03 d (1.6)	4.92 d (1.7)	5.11 d (1.8)
15	5.52 d (2.1)	5.66 dd (1.5; 0.8)	5.66 dd (0)	3.34 d (4.2)
15′	5.44 d (1.8)	5.47 dd (1.7; 0.8)	5.47 m	3.07 d (4.2)
8-OH		1.85 brs	1.85 brs	
2′	2.40 m	2.42 m	2.48 m	-
3′	1.67 m; 1.48 m	1.63 m; 1.51 m	1.17 d	3.88 d
4′	0.90 m	1.15 d	1.16 d	1.55 s
5′	1.16 q	0.69 m	-	-

^a^ in CDCl_3_; ^b^ in CD_3_OD; dd—a doublet of doublets; d—doublet; m—multiplet; brs—broaden singlet; s—singlet; q—quartet.

**Table 2:** The results of the antifungal activity of the examined compounds from *P. bellus* (growth inhibition zone (mm) ± standard deviation).

	Name of Fungal Strains																			
Compound	*Candida albicans*		*Candida famata*		*Candida glabrata*		*Candida parapsilosis*		*Rhodotorula rubra*		*Trichophyton rubrum*		*Trichophyton mentagrophytes var. interdigitale*		*Trichophyton mentagrophytes var. interdigitale*		*Microsporum canis*		*Scopulariopsis brevicaulis*	
	**G**	**I**	**G**	**I**	**G**	**I**	**G**	**I**	**G**	**I**	**G**	**I**	**G**	**I**	**G**	**I**	**G**	**I**	**G**	**I**
Acroptilin (13)	yes	no	yes	6.25 ± 0.35	yes	no	yes	no	yes	no	yes	21.5 ± 0.71	yes	6.5 ± 0.71	yes	6.25 ± 0.35	yes	no	yes	no
Cebellin A (11)	yes	no	X	X	yes	18.5 ± 0.71	X	X	yes	11.75 ± 0.35	no	no	yes	15.5 ± 0.71	no	no	yes	no	yes	10.5 ± 0.71
Cebellin B (12)	yes	no		10.5 ± 0.71	yes	12.5 ± 0.71	yes	8.25 ± 0.35	yes	no	yes	12.25 ± 0.35	yes	10.25 ± 0.35	yes	12.25 ± 0.35	yes	no	yes	8.5 ± 0.71
Cebellin L (1)	yes	no	yes	15.5 ± 0.71	yes	14.25 ± 0.35	yes	26.5 ± 0.71	yes	no	yes	12.5 ± 0.71	yes	20.5 ± 0.71	yes	18.5 ± 0.71	yes	no	yes	10.5 ± 0.71
Cebellin K + N + O (2-4)	yes	12.25 ± 0.35	X	X	yes	19.75 ± 0.35	X	X	yes	21.5 ± 0.71	no	no	yes	24.25 ± 0.35	no	no	yes	20.25 ± 0.35	yes	9.25 ± 0.35
Full extract of *P. bellus*	yes	no	yes	12	yes	12.5 ± 0.71	yes	4 ± 1.41		no	yes	24.5 ± 0.71	yes	18.5 ± 0.71	yes	33.5 ± 0.71	yes	no	yes	no

G, growth of fungal strain; I, inhibition of fungal strain growth; X, not examined. The results are expressed as growth inhibition zone (mm) ± standard deviation (SD).

**Table 3:** The results of the difference test between averages for the examined compounds in relation to various fungal strains.

Compound		*Candida famata*	*Candida glabrata*	*Candida parapsilosis*	*Rhodotorula rubra*	*Trichophyton rubrum*	*Trichophyton mentagrophytes* var *interdigitale*	*Scopulariopsis brevicaulis*
		*p*-Value						
**Acroptilin** **(13)**	Cebellin A (11)	-	-	-	-	-	0.0061 *	-
	Cebellin B (12)	0.0169 *	-	-	-	0.0036 *	0.0216 *	-
	Cebellin L (1)	0.0036 *	-	-	-	0.0062 *	0.0026 *	-
	Cebellin K + N + O (2–4)	-	-	-	-	-	0.001 *	-
	Full extract of *P. bellus*	-	-	-	-	0.0517	0.0035 *	-
**Cebellin A** **(11)**	Cebellin B (12)	-	0.0137 *	-	-	-	0.0112 *	0.1063
	Cebellin L (1)	-	0.0169 *	-	-	-	0.0196 *	1
	Cebellin K + N + O (2–4)	-	0.1552	-	0.0033 *	-	0.0041 *	0.1552
	Full extract of *P. bellus*	-	0.0137 *	-	-	-	0.0517	-
**Cebellin B** **(12)**	Cebellin L (1)	0.0196 *	0.0889	0.0009 *	-	0.6988	0.003 *	0.1063
	Cebellin K + N + O (2–4)	-	0.0059 *	-	-	-	0.0016 *	0.3122
	Full extract of *P. bellus*	-	1	0.0531	-	0.0021 *	0.0046 *	-
**Cebellin L** **(1)**	Cebellin K + N + O (2–4)	-	0.004 *	-	-	-	0.0216 *	0.1552
	Full extract of *P. bellus*	-	0.0889	0.002 *	-	0.0035 *	0.1063	-
**Cebellin K + N + O** **(2–4)**	Full extract of *P. bellus*	-	0.0059 *	-	-	-	0.0093 *	-

*—statistically significant (*p* < 0.05).

## Data Availability

Data is contained within the article.
